# Expression of Erk5 in Early Stage Breast Cancer and Association with Disease Free Survival Identifies this Kinase as a Potential Therapeutic Target

**DOI:** 10.1371/journal.pone.0005565

**Published:** 2009-05-15

**Authors:** Juan Carlos Montero, Alberto Ocaña, Mar Abad, María Jesús Ortiz-Ruiz, Atanasio Pandiella, Azucena Esparís-Ogando

**Affiliations:** 1 Instituto de Biología Molecular y Celular del Cáncer, CSIC-Universidad de Salamanca, Salamanca, Spain; 2 Oncology Service and AECC Unit, Complejo Hospitalario Universitario de Albacete, Albacete, Spain; 3 Department of Pathology, Hospital Universitario de Salamanca, Salamanca, Spain; Ordway Research Institute, United States of America

## Abstract

**Background:**

Breast cancer is the most common neoplasia in women. Even though advances in its treatment have improved disease outcome, some patients relapse. Therefore, attempts to better define the molecular determinants that drive breast cancer cell proliferation may help in defining potential therapeutic targets. Mitogen-activated protein kinases (MAPK) play important roles in tumorigenesis. One of them, Erk5, has been linked to the proliferation of breast cancer cells *in vitro*. Here we have investigated the expression and prognostic value of Erk5 in human breast cancer.

**Methodology/Principal Findings:**

Animal and cellular models were used to study Erk5 expression and function in breast cancer. In 84 human breast tumours the expression of Erk5 was analyzed by immunohistochemistry. Active Erk5 (pErk5) was studied by Western blotting. Correlation of Erk5 with clinicopathological parameters and with disease-free survival in early stage breast cancer patients was analyzed. Expression of Erk5 was detected in most patients, and overexpression was found in 20%. Active Erk5 was present in a substantial number of samples, as well as in tumours from an animal breast cancer model. Overexpression of Erk5 was associated with a decrease in disease-free survival time, which was independent of other clinicopathological parameters of prognosis. Transient transfection of a short hairpin RNA (shRNA) targeting Erk5, and a stable cell line expressing a dominant negative form of Erk5 (Erk5^AEF^), were used to investigate the influence of Erk5 on drugs used in the clinic to treat breast tumours. We found that inhibition of Erk5 decreased cancer cell proliferation and also sensitized these cells to the action of anti-HER2 therapies.

**Conclusions/Significance:**

Overexpression of Erk5 is an independent predictor of disease-free survival in breast cancer, and may represent a future therapeutic target.

## Introduction

Breast cancer represents the most common cancer type in women [1], [2]. Although important advances in the treatment of this disease have improved patient survival, its disseminated metastatic form still remains incurable. Furthermore, a number of early stage breast cancer patients will relapse regardless of the adjuvant treatment given. Because of this, substantial efforts have been made to better understand the molecular bases of the disease in order to achieve more efficient treatments. These studies led to the identification of certain oncogenic stimuli linked to the genesis and/or progression of breast cancer. One of these oncogenic proteins is the transmembrane tyrosine kinase HER2 [3]. This kinase belongs to the family of mammalian HER/ErbB receptors, that includes the epidermal growth factor receptor (EGFR or HER1), HER2/ErbB2, HER3/ErbB3, and HER4/ErbB4 [4]. Overexpression of HER2 occurs in 20–30% of breast cancer tumours, and is linked to a more aggressive phenotype and a worse prognosis [3]. The identification of HER2 as an important oncogene in breast cancer led to the development of anti-HER2 therapies such as the monoclonal antibody trastuzumab (Herceptin®) or the small tyrosine kinase inhibitor lapatinib. Treatment with these agents has demonstrated efficacy in breast cancer patients overexpressing HER2 [5]–[7].

Tumorigenic signalling from HER receptors involves the activation of several signal transduction routes. Among the most important are the Mitogen-Activated Protein Kinases (MAPK) and the Phosphatidylinositol-3-kinase (PI3K)/Akt routes [4]. Four major MAPK routes have been described [8]. The Erk1/2 pathway is activated by growth factor receptors and transduces proliferation signals. Another two MAPK routes, the p38 and Jun kinase pathways, are activated by several cytokines and stress stimuli, and have been mainly linked to the regulation of apoptotic cell death [9]. Recently, a novel MAPK termed Erk5 has also been involved in the control of cellular proliferation and cell death, as well as in tumorigenesis [10]. Elegant *in vivo* studies using animals in which Erk5 expression can be regulated, have demonstrated that Erk5 is important for sustaining tumour growth, probably due to its supportive role in vasculogenesis and blood vessel homeostasis [11], [12]. In the breast, Erk5 activation has been linked to growth factor-induced proliferation of normal breast epithelial cells [13] as well as breast cancer cells [13], [14]. In the latter, activation of HER receptors by the ligand Neuregulin (NRG) provoked Erk5 activation [14]. Furthermore, restriction of Erk5 activation by expression of a dominant negative inhibitory form partially blocked proliferation of breast cancer cells [14]. Interestingly, *in vitro* studies showed that Erk5 activity is constitutively high in breast cancer cell lines overexpressing HER2 [14].

These precedents support a potential role of Erk5 in breast cancer initiation/progression. We therefore decided to explore the *in vivo* expression and activation status of Erk5, its potential association to HER2 overexpression, and prognostic relevance in breast cancer. We show that Erk5 is overexpressed in the tumours of a number of breast cancer patients. Moreover, our findings indicate that Erk5 overexpression is an independent prognostic marker for disease-free survival. In addition, *in vitro* studies indicated that inhibition of Erk5 sensitized cells to treatments commonly used in the breast cancer clinic. Therefore, Erk5 may represent a new prognostic marker in breast cancer, and could also represent a novel therapeutic target.

## Materials and Methods

### Patient samples and immunohistochemistry

During the period from 1999 to 2005 a total of 84 breast tumours were randomly obtained from the Pathology Department of the Salamanca University Hospital (Salamanca, Spain). All patients provided written informed consent for the collection of samples and subsequent analysis. The procedures were approved by the Institutional Review Board Ethics Committee on Human Research of the Salamanca University Hospital. The clinicopathological characteristics of these patients are described in Table 1.

**Table 1:** Patient and Tumour Characteristics (n = 84).

Patient age (n = 81)		median 58 (range 31–89)
Grade (n = 77)	1–2	32
	3	45
Tumour diameter (mm) (n = 72)		median 26.5 (range 8–75 mm)
Estrogen Receptors (n = 84)	Positive	52
	Negative	32
Progesterone Receptors (n = 84)	Positive	49
	Negative	35
HER2 FISH (n = 84)	Positive	24
	Negative	60
Lymph nodes (n = 77)	Positive	51
	Negative	26

HER2 was analyzed using the Dako Herceptest kit (DakoCytomation, Carpinteria, CA). Erk5 expression was analyzed using an affinity-purified antibody, that specifically recognizes the C-terminus of Erk5 (residues 781–805) [14]. That this antibody specifically detected Erk5 in immunohistochemistry was indicated by the prevention of the Erk5 staining upon preincubation of the antibody with the peptide against which the antibody had been raised (data not shown, see also ref. [14]). For the clinicopathological studies, Erk5 expresion levels were scored only in the epithelial tumoral cells. A quantitative analysis was based on the percentage of stained cells and intensity of the staining, and was defined as follows: 0, no appreciable staining in cells (0 to <10%); 1, weak intensity in cells (between 10% and 30%); 2, intermediate intensity of staining (30% to 60%); and 3, strong intensity of staining (>60%). Normal breast stained with an intensity that scored as 1. Tumours were considered positive (Erk5 high) when scored 2 or 3, and negative when scored 0 or 1. HER2 gene amplification (evaluated by FISH DAKO HER2 FISH pharmDx™ Kit, DakoCytomation, Glostrup, Denmark A/S) was defined as a HER2-chromosome 17 ratio of >2.0, as required by the guidelines.

### Statistical analyses

Statistical analyses were performed using the SPSS Data Analysis Program, version 13.0 (SPSS, Inc., Chicago, IL). Association between two dichotomous variables was determined using the chi-square test and two tailed Fisher's exact test. Spearman's nonparametric correlation test was used to calculate the statistical significance of continuous variables. Mann-Whitney U test was used to compare continuous variables with dichotomic or ordinal variables. Disease Free Survival (DFS) was calculated from the date of diagnosis to the date of recurrence or death. Patients who were event free at the date of last follow-up were censored at that time. Kaplan-Meier survival analyses were carried out for DFS. Differences in DFS according to Erk5 expression were compared using the Log-rank test. Multivariate analysis using the Cox proportional hazards models was performed to define prognostic independent factors for DFS. All statistical tests were conducted at a two-sided significance level of 0.05.

### Cell culture and transfections

The cell line BT474 was cultured at 37°C in a humidified atmosphere in the presence of 5% CO2–95% air. Cells were grown in Dulbecco's modified Eagle medium (DMEM) containing a high glucose concentration (4,500 mg/liter) and antibiotics (penicillin at 100 U/ml, streptomycin at 100 µg/ml) and supplemented with 10% foetal bovine serum (FBS).

Transfections of BT474 cells with pSuper or pSuper-shErk5 [15] were performed using LipofectAMINE (Invitrogen, San Diego, CA) following the manufacturer's instructions. BT474 cells that express HA-Erk5^AEF^ have been described [14].

### Cell proliferation measurements

Subconfluent monolayer cultures were trypsinized, and cells were plated in 24-well plates at a density of 20,000 per well. Cultures were allowed to attach overnight, and the medium was then replaced with medium containing lapatinib 100 nM or trastuzumab 10 nM. Cell proliferation was analyzed at 3 days by an MTT-based assay [14]. Briefly, the medium in each well was replaced with 250 µl of fresh medium containing MTT at 0.5 µg/µl and plates were returned to the incubator for 1 h. The medium-MTT was then removed, 500 µl of dimethyl sulfoxide was added to each well, and the plate was kept in agitation for 5 min in the dark to dissolve the MTT-formazan crystals. The absorbance of the samples was then recorded at 570 nm. Four wells were analyzed for each condition, and wells containing medium plus MTT but no cells were used as blanks. The results are presented as the mean ± the standard deviation (SD) of quadruplicates of a representative experiment that was repeated at least three times.

### FVB/neu mice tumour samples

For the *in vivo* analyses in mice, the FVB parental and FVB/N-Tg (MMTVneu) 202 Mul/J strains (The Jackson Laboratory, Bar Harbor, Maine) were manipulated at the animal facility (Servicio de Experimentación Animal de la Universidad de Salamanca, code: PAE/SA/001) following the European legal and institutional guidelines (86/609/CEE). Tumour growth was evaluated after a period of six months after birth, and tumour samples were obtained after sacrifice of the animals, and immediately frozen in liquid Nitrogen.

### Western blotting experiments

Frozen human and mice tumoral samples were inspected by haematoxylin-eosin staining for epithelial tumour content by analysis of two slices at each end of the tumour. Only samples containing >70% epithelial tumoral cells were selected for Western analyses. The tumours were minced, washed with phosphate-buffered saline (PBS), and homogenized in ice-cold lysis buffer (140 mM NaCl; 10 mM EDTA; 10% glycerol; 2% Triton X-100; 20 mM Tris pH 7.0; pepstatin, 10 µM; aprotinin, 10 µg/ml; leupeptin, 10 µg/ml; PMSF, 1 mM; beta-glycerophosphate, 25 mM; sodium fluoride, 10 mM; and sodium orthovanadate, 10 mM) with a tight-fitting Dounce homogenizer [16]. This homogenate was centrifuged at 10,000×*g* for 20 minutes at 4°C, and the supernatants were transferred to new tubes. BT474 cell extracts were prepared as described [14]. The samples (0.5 mg, measured by the Bradford assay) were immunoprecipitated at 4°C for at least 2 h with either the anti-Erk5 (1 µl) or the anti-pErk5 (10 µl) antibody and the immune complexes were recovered by a short centrifugation, followed by three washes with 1 ml of cold lysis buffer. Samples were then boiled in electrophoresis sample buffer and loaded onto SDS-PAGE gels. After transfer to polyvinylidene difluoride membranes, filters were blocked for 1 h in Tris buffered saline with 0.2% Tween 20 (TBST) and then incubated for 2 to 16 h with the corresponding antibody. After being washed with TBST, filters were incubated with horseradish peroxidase-conjugated secondary antibodies for 30 min and bands were visualized by a luminal-based detection system with *p*-iodophenol enhancement [17].

Quantitation of Erk5, pErk5, HER2, pHER2 and pancytokeratins in Westerns was performed using the NIH Image 1.61 program. HER2 and pHER2 were analyzed as described [16]. The antibodies for calnexin or GAPDH, that we used as controls for protein loading, were from Stressgen (Victoria, BC, Canada) or Santa Cruz Biotechnology Inc (Santa Cruz, CA), respectively. The anti-pancytokeratin antibody was from DakoCytomation (Carpinteria, CA).

## Results

### Expression of Erk5 in human breast cancer

**Figure 1:**
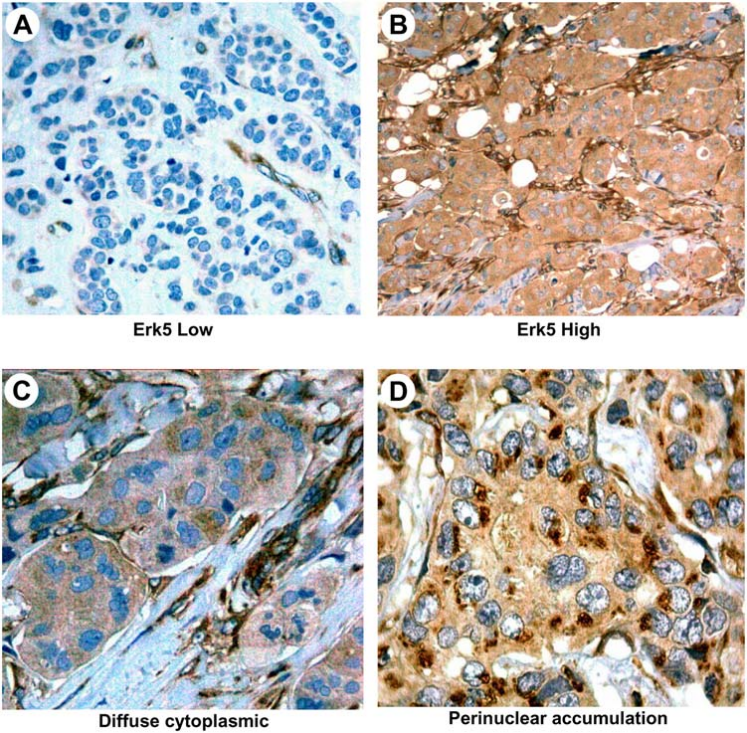
Patterns of Erk5 staining in human breast cancer. Immunohistochemical staining of Erk5, showing samples scored as low (panel *A*), or high expression level (panel *B*). Two patterns of Erk5 staining in breast cancer are shown in panels *C* and *D*. Magnification: for A and B, 200×; for C and D, 1000×.

Erk5 expression was analyzed by immunohistochemistry in paraffin samples from 84 primary breast cancer tumours. For these analyses, we used an affinity purified anti-Erk5 antibody that recognizes the C-terminal region of Erk5 [14]. Using a two-tier distribution (low/high) in which values equal to or above 2 were considered positive (see Materials and Methods for details and Figure 1A and B), high levels of Erk5 in the epithelial tumour cells were observed in 17 patients (20% of the patients). We observed two patterns of Erk5 staining. One corresponded to a diffuse cytoplasmic signal (Figure 1C), and a second pattern was represented by a perinuclear cytoplasmic accumulation of Erk5 (Figure 1D). Staining of Erk5 was also observed in the majority of the endothelial cells and stromal fibroblasts. The staining of endothelial cells may be related to the proposed role of Erk5 in neoangiogenesis [11], [12].

**Figure 2:**
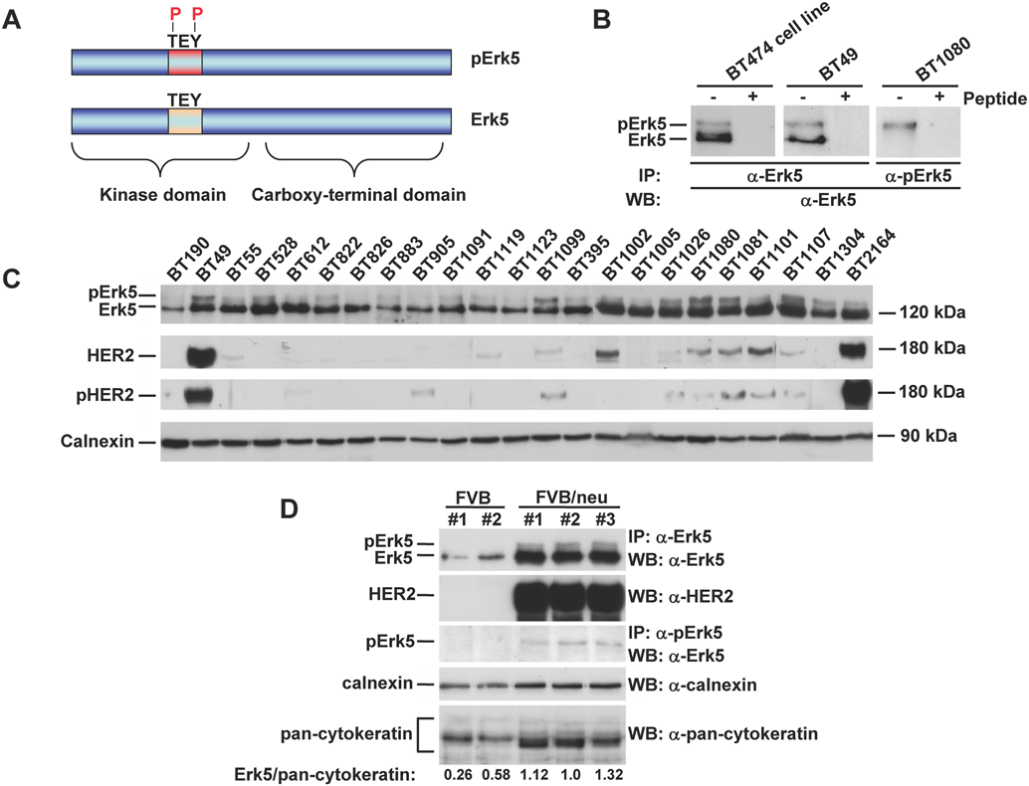
Molecular forms of Erk5 in human breast cancer. *A*, Schematic representation of Erk5 depicting the region where it is dually phosphorylated at the TEY microdomain. *B*, Identification of the bands recognized by the anti-Erk5 antibodies in BT474 cells, and in the breast cancer samples BT49, and BT1080. The immunoprecipitates and Westerns were probed with the indicated antibodies. Some samples were immunoprecipitated with the antibodies but in the presence of an excess of the peptide used for the raising of the antibody, the peptide excess prevented immunoprecipitation of both Erk5 bands. *C*, Molecular forms of Erk5 detected in breast cancer samples, and expression of HER2 and pHER2. Calnexin was used as a control for protein loading. *D*, Expression of Erk5, pErk5, and HER2 in the breast of transgenic (FVB/neu) tumoral breast tissue, or normal (FVB) breast tissue from mice. The amount of epithelial cytokeratins is shown.

In addition to the study of overall Erk5 expression, we also analyzed the degree of activation of Erk5. The mechanism of activation of Erk5, as other MAPKs, involves dual phosphorylation of Erk5 at the Thr-Glu-Tyr microdomain that is present in its activation loop (Figure 2A) [18]. Due to the lack of a specific antibody that would only detect active Erk5 in immunohistochemical analyses, we analyzed the level of active, dually phosphorylated Erk5 by Western blotting. This activation can be followed by either using an antibody that uniquely recognizes double phosphorylated Erk5 (anti-pErk5), or by the anti-C-terminus antibody described above that recognizes both Erk5 and pErk5 [14]. As pErk5 migrates slower than Erk5, both forms can be differentiated in Western blotting experiments by their different mobilities. Initially, we used two patient samples to characterize the reaction of these antibodies on human tissue-derived material; and used as a control BT474 cells which express both Erk5 and pErk5 [14] (Figure 2B). The anti-Erk5 antibody precipitated two Erk5 forms in the BT49 patient sample (Figure 2B). When the BT49 immunoprecipitates were performed in the presence of an excess of the peptide against which the anti-Erk5 C-terminal antibody was raised, no Erk5 bands were detected, indicating that in fact both bands corresponded to Erk5. Analogously, the anti-pErk5 antibody specifically precipitated the upper band in extracts from the BT1080 tumoral tissue, indicating that this band corresponded to pErk5. The amount of Erk5 and pErk5 was then analyzed on samples of patients from which frozen tissue was available (n = 23). Erk5 was detected in all the samples; however, its amount varied among distinct patient samples. In a substantial number of the samples analyzed a band with a retarded migration with respect to Erk5, that corresponded to pErk5, was observed (Figure 2C). We took these data to indicate that Erk5 activation is frequent in breast cancer.

### HER2 expression and Erk5 activation

As activation of Erk5 may be caused by activation of HER2 receptors in breast cancer *in vitro* [14], we analyzed whether such a relationship also existed *in vivo*. To this end we used a mouse model of breast cancer in which overexpression of HER2 provokes mammary gland tumorigenesis [19]. Tumours resected from these mice contained increased amounts of Erk5, pErk5, and HER2, compared to normal breast tissue from the non-transgenic strain (Figure 2D). The Erk5/pancytokeratin ratio present in the tumoral tissue was higher than in normal breast, suggesting that in this breast cancer model Erk5 was overexpressed in the epithelial tumoral cells. Therefore, increased levels of HER2 may cause increased expression of Erk5, and may favour its activation.

Immunohistochemical analyses of HER2 and Erk5 expression in the patient series showed a trend towards a positive correlation between high expression level of Erk5 and HER2 positivity (*p* = 0.074). Interestingly, in Western blot analysis from patients samples, and even though in some tumours pErk5 accompanied HER2 overexpression (e.gr. BT49), other tumours in which HER2 was not overexpressed also contained pErk5 (e.gr. BT1304), indicating that Erk5 may also be activated by routes other than HER2 overexpression.

### Association of Erk5 with prognosis

We reviewed the clinical characteristics of our series of patients and studied if some of these characteristics such as age, tumour grade, tumour size, hormone receptor status and axillary lymph nodes, correlated with their amount of immunohistochemical tumour Erk5. No correlation was observed for any of these parameters, with the exception of a positive association with the expression of the progesterone receptor (*p* = 0.024) (Table 2).

**Table 2:** Association between Clinicopathologic Variables and Erk5 Expression.

*Clinicopathologic variables*	*p*
Age	0.156*
Tumour grade	0.608*
Tumour size	0.201*
Estrogen Receptors	0.145 #
Progesterone Receptors	0.024 # †
Axillary lymph nodes	0.078 #

* Mann-Whitney U test.

# Fisher's exact test.

† Statistically significant.

**Figure 3:**
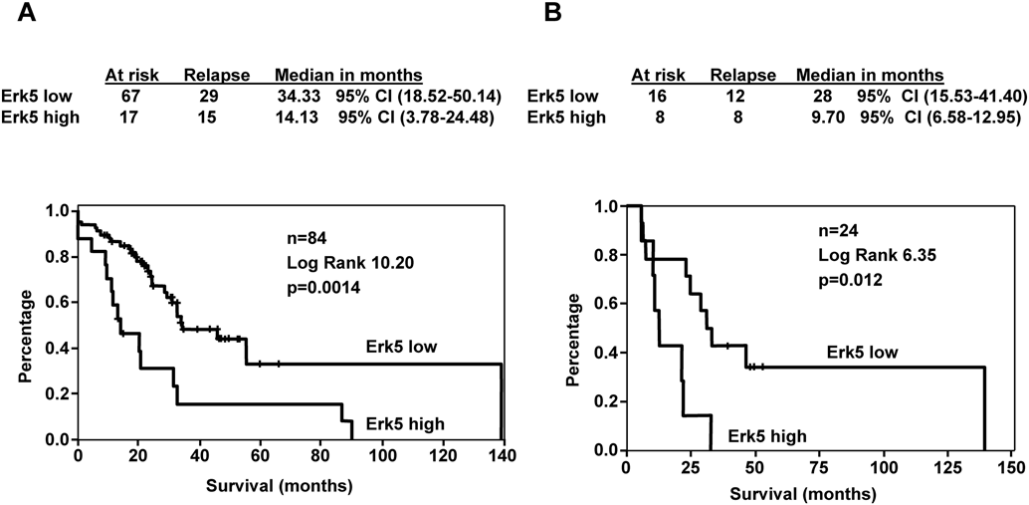
Relationship between Erk5 expression and disease-free survival. *A*, Kaplan-Meier plots of disease-free survival with respect to Erk5 levels in 84 early stage breast cancer patients. Patients with high levels of Erk5 (n = 17) had a worse disease-free survival (DFS) time (14.13 months; 95% CI: 3.78–24.48) compared with patients (n = 67) with low levels (34.33 months; 95% CI: 18.52–50.14). The difference was statistically significant in the log-rank test (*p* = 0.0014). *B*, Kaplan-Meier plots of disease-free survival with respect to Erk5 levels in 24 HER2 positive early stage breast cancer patients. Patients with high levels of Erk5 (n = 8) had a worse disease-free survival (DFS) time (9.70 months; 95% CI: 6.58–12.95) compared to patients (n = 16) with low levels (28 months; 95% CI: 15.53–41.40). The difference was statistically significant in the log-rank test (*p* = 0.012).

To explore if Erk5 was associated with a more aggressive phenotype, we studied the prognosis of these patients with respect to the immunohistochemical Erk5 levels. Importantly, patients with high levels of Erk5 (n = 17) had a worse disease free survival (DFS) time compared with the patients with low levels (n = 67) (log-rank test 10.20, *p* = 0.0014) (Figure 3A). Median DFS time was 34.33 (95% CI: 18.52–50.14) months for patients with low Erk5 levels compared with 14.13 (95% CI: 3.78–24.48) months for patients with high levels. An univariate analysis was performed to study variables that could be associated with prognosis (Table 3). In this analysis only oestrogen receptors, HER2 status by FISH, and Erk5 were prognostic factors. A further multivariate analysis using a Cox Regression model including the univariate prognostic factors and those with clinical relevance in breast cancer, showed that Erk5 was an independent prognostic factor (Table 4). The influence of Erk5 on DFS was clearly demonstrated when HER2 positive and HER2 negative patients were analyzed separately (Figure 3B and data not shown). Figure 3B shows the Kaplan-Meier curve for HER2 positive patients regarding Erk5 expression. As can be seen, patients with Erk5 positive tumours have a worse DFS compared with the negative ones. These findings suggest that Erk5 may be a new biomarker associated with worse outcome in breast cancer.

**Table 3:** Association of Erk5 with Disease Free Survival in 84 Breast Cancer Patients Using the Univariate Proportional Hazards Model (Cox Analisis).

	Relative risk	95% CI	*p*
Erk5 Low expression vs. high	2.786	(1.389–5.587)	0.004
HER2 FISH amplification vs. no amplification	1.935	(1.011–3.704)	0.046
Tumour size <3 vs. >3 cm	2.035	(0.967–4.280)	0.061
Axillary lymph nodes Positive vs. negative	1.821	(0.721–4.600)	0.205
Tumour Grade 1–2 vs. 3	1.663	(0.765–3.618)	0.199
Estrogen Receptors Positive vs. negative	0.421	(0.214–0.832)	0.013
Progesterone Receptors Positive vs. negative	0.580	(0.285–1.180)	0.133
Age <60 vs. >60 years	0.627	(0.309–1.274)	0.197

**Table 4:** Prognostic Factors in 84 Breast Cancer Patients Using the Backward Selection Model of the Cox Regression.

	Relative risk	95% CI	*p*
Erk5 Low expression vs. high	3.799	(1.488–9.703)	0.005
HER2 FISH Amplification vs. no amplification	1.364	(0.371–5.009)	0.640
Tumour size <3 vs. >3 cm	2.141	(0.727–6.304)	0.167
Axillary lymph nodes Positive vs. negative	1.131	(0.275–4.651)	0.865
Tumour Grade 1–2 vs. 3	0.620	(0.155–2.480)	0.499
Estrogen Receptors Positive vs. negative	0.460	(0.120–1.177)	0.259
Progesterone Receptors Positive vs. negative	1.894	(0.486–7.379)	0.358
Age <60 vs. >60 years	0.650	(0.192–1.907)	0.391

### Erk5 regulates the response to anti-HER2 treatments

**Figure 4:**
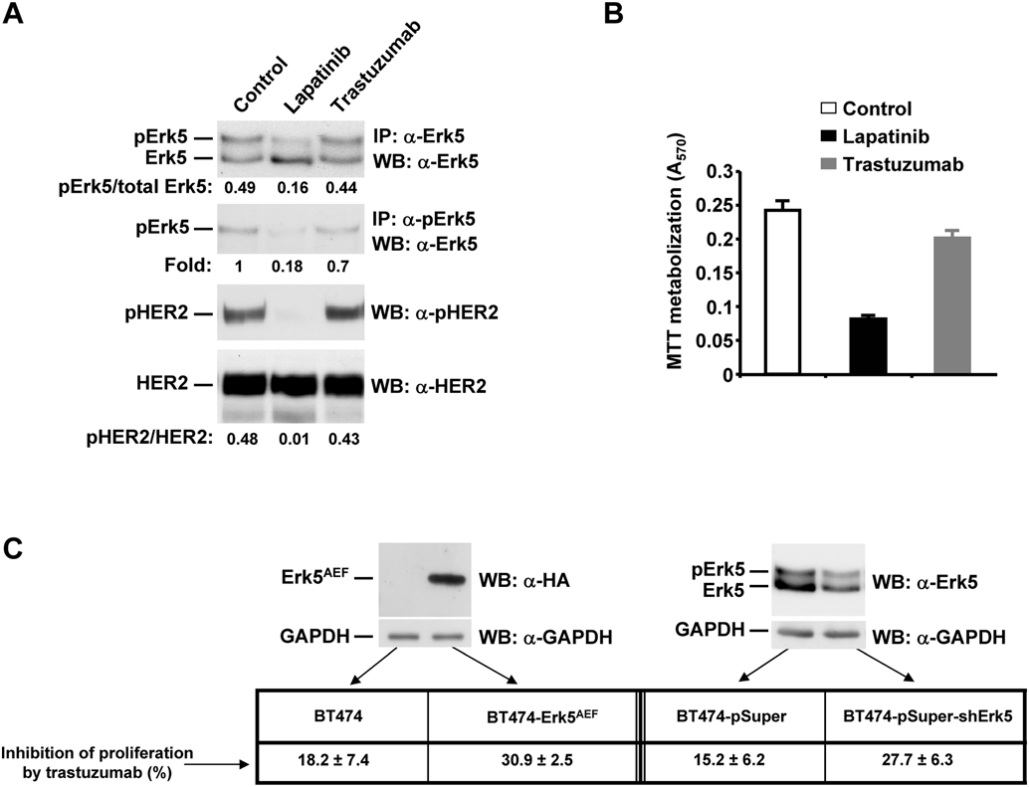
Inhibition of Erk5 sensitizes to the action of Trastuzumab. *A*, Action of lapatinib and trastuzumab on Erk5, pErk5, and pHER2 in BT474 cells. The drugs were added for 24 hours and then cell extracts prepared to be immunoprecipitated with anti-Erk5, anti-pErk5, or anti-HER2. *B*, Effect of lapatinib (100 nM) or trastuzumab (10 nM) on the MTT uptake of BT474 cells. The results are reported as the mean±SD of quadruplicates. *C*, Action of Erk5^AEF^ or Erk5 shRNA on cell proliferation. Erk5^AEF^ expression was measured by Western blot using an anti-HA tag antibody, as the Erk5^AEF^ form is tagged with an HA epitope. GAPDH was used as a control for protein loading. BT474 cells transfected with the indicated vectors were treated with trastuzumab and their proliferation measured using MTT uptake.

The negative impact of Erk5 overexpression on patient outcome led us to analyze whether Erk5/pErk5 expression regulated the response to treatments used in the breast cancer clinic. Since the results obtained with the HER2 transgenic model together with the analyses in patient samples indicated a relationship between HER2 and Erk5/pErk5 levels, we investigated whether treatment with agents that target HER2 had an effect on the levels or activity of Erk5. BT474 cells, that express constitutively active HER2 and Erk5 [14], were treated with the small molecule inhibitor of HER2 lapatinib, or trastuzumab. Treatment with lapatinib strongly inhibited HER2 tyrosine phosphorylation, and decreased the level of pErk5 (Figure 4A). Trastuzumab had a minimal effect on the pErk5 level, even though a small decrease was observed when quantitative analyses were performed using the anti-Erk5 or anti-pErk5 antibodies. Quantitative analyses of the pHER2/HER2 ratios indicated that trastuzumab had a very weak effect on HER2 activation. Concurrent cell proliferation studies indicated that lapatinib reduced the proliferation of BT474 cells to a greater extent than trastuzumab (Figure 4B).

The relative partial resistance to trastuzumab led us to investigate whether decreasing Erk5 function could facilitate trastuzumab action. We used two different strategies, one aimed at decreasing Erk5 function by expressing a dominant negative form; and another aimed at decreasing Erk5 levels using RNAi. The dominant negative form of Erk5 (Erk5^AEF^) acts by competing with endogenous Erk5, impairing the activation of the latter [14]. Biochemical studies confirmed that BT474- Erk5^AEF^ expressed the dominant negative form, and the RNAi strategy decreased Erk5/pErk5 levels (Figure 4C). A three day treatment of BT474 cells with trastuzumab decreased their proliferation by 18.2±7.4% (mean±SD). In BT474-Erk5^AEF^ cells trastuzumab decreased proliferation by 30.9±2.5%. In BT474 cells transfected with the empty vector pSuper used for the RNAi experiments, trastuzumab decreased their proliferation by 15.2±6.2%. In BT474 cells transfected with the vector containing the Erk5 interference sequence, trastuzumab decreased their proliferation by 27.7±6.3%. Therefore, treatments that decrease Erk5/pErk5 levels facilitate the antitumoral action of trastuzumab.

## Discussion

This study was initiated on the basis of previous findings from our group that showed a role of Erk5 in the control of breast cancer cell proliferation [14]. In those studies, Erk5 was activated by the HER ligand NRG, and interference with Erk5 activation resulted in restricted cell proliferation [14]. Furthermore, in breast cancer cells overexpressing HER2, Erk5 was constitutively activated. Here we have studied Erk5 expression and relevance in human breast cancer. Pathologic analyses indicated that Erk5 was overexpressed in 20% of patients with breast cancer. Therefore, increased expression of Erk5 may accompany breast cancer tumorigenesis in certain instances. Immunohistochemical staining also showed expression of Erk5 in stromal tissue, as well as in blood vessel endothelial cells. As Erk5 has been implicated in tumour angiogenesis, its presence in the tumour vessels points to a role of this kinase in the construction of the neovasculature of the tumour, and may thus be considered a potential target for therapeutic intervention.

As Erk5 has been shown to participate in proliferative signalling upon activation of HER2 [14], we explored whether a correlation existed between Erk5 expression levels or activation and HER2 expression in the patient series. With respect to the expression levels, a trend was found between high level of expression of immunohistochemical Erk5 and HER2 positivity. Furthermore, tumours from mice in which overexpression of HER2 provokes mammary gland tumorigenesis contained elevated levels of Erk5 when compared to normal tissue from non-transgenic animals. These results indicate a certain degree of linkage between HER2 and Erk5 overexpression.

The frequent expression of pErk5 in breast tumours merits some comments. Detectable levels of pErk5, as analyzed by Western blotting experiments, were observed in most of the patient samples. Given the precedents that link Erk5 activation to cell proliferation [10], it is possible that expression of active Erk5 may contribute tumour proliferation. One aspect that deserves attention is the fact that our Western assays measured the pErk5 signal in the whole tumoral mass, which includes the epithelial as well as the stromal tumoral tissue. It is therefore possible that a fraction of the Erk5 and pErk5 signal may derive from the stromal tumoral tissue. Noteworthy, we observed that the complement of Erk5 in the stromal tumoral tissue as analyzed by immunohistochemistry was variable between samples from different patients (e. gr. Figure 1). This is important, and supports the increasingly accepted concept that the stromal tissue that is part of the tumour may phenotypically be distinct from the normal breast stroma. With respect to the level of pErk5 and HER2, several evidences indicate that activation of Erk5 may occur by HER2 overexpression but also through other mechanisms. In support of a linkage between HER2 and pErk5 are the data obtained from the mouse model we used, in which HER2-driven tumorigenesis is accompanied by pErk5, and the fact that tumour patient samples with HER2 overexpression and phospho-HER2 expression also expressed pErk5. However, other patient samples presented pErk5 even though no overexpression of HER2 was detected. Indeed, pErk5 was present more frequently than HER2 overexpression. This is interesting as it indicates that Erk5/pErk5 levels may also be regulated by HER2-independent routes. In support of this conclusion is also the fact that treatment of BT474 cells with lapatinib, that fully inhibited HER2 phosphorylation, only partially inhibited pErk5 levels. Moreover, other membrane receptors such as, IL6 receptor [20], or cytosolic kinases such as Src [21], have also been reported to active Erk5.

A clinically relevant finding of our work is the linkage of Erk5 expression level to patient outcome. Thus, patients with high levels of Erk5 relapsed earlier than patients with low Erk5. Statistically, the expression level of this kinase was independent of other prognostic factors, as was demonstrated by the multivariate analyses. The predictive nature of Erk5 expression with respect to disease relapse was also confirmed by using a selected set of patients characterized by HER2 overexpresion. These findings suggest that Erk5 may represent a potential interesting target. Moreover, the fact, that *in vitro* reduction of Erk5 activation or expression favoured the action of trastuzumab indicates that targeting of Erk5 may facilitate the action of anti-HER2 therapies and may also help in reverting drug resistance. With respect to the latter, it will be interesting to define whether trastuzumab resistance is accompanied by increased levels of Erk5 or pErk5.

In conclusion, our findings indicate that Erk5 may have an important role in breast cancer, and its overexpression predicts patient outcome. Furthermore, as Erk5 is expressed in tumoral as well as other cellular components of the tumour, such as stromal fibroblasts or endothelial cells, targeting of this kinase may be of therapeutic benefit in breast cancer.
